# Ginseng Gintonin Attenuates Lead-Induced Rat Cerebellar Impairments during Gestation and Lactation

**DOI:** 10.3390/biom10030385

**Published:** 2020-03-02

**Authors:** Sung Min Nam, Sun-Hye Choi, Hee-Jung Cho, Jin Seok Seo, Minsuk Choi, Sang-Soep Nahm, Byung-Joon Chang, Seung-Yeol Nah

**Affiliations:** 1 Department of Anatomy, College of Veterinary Medicine, Konkuk University, Seoul 05029, Korea; skavet@konkuk.ac.kr (S.M.N.); phoenix_1st@naver.com (J.S.S.); xcv3bn@naver.com (M.C.); ssnahm@konkuk.ac.kr (S.-S.N.); bjchang@konkuk.ac.kr (B.-J.C.); 2 Ginsentology Research Laboratory and Department of Physiology, College of Veterinary Medicine, Konkuk University, Seoul 05029, Korea; vettman@konkuk.ac.kr (S.-H.C.); ouddi80@naver.com (H.-J.C.)

**Keywords:** ginseng, gintonin, pregnancy and lactation, cerebellar lead (Pb) poisoning, neuroprotection

## Abstract

Gintonin, a novel ginseng-derived lysophosphatidic acid receptor ligand, improves brain functions and protects neurons from oxidative stress. However, little is known about the effects of gintonin against Pb-induced brain maldevelopment. We investigated the protective effects of gintonin on the developing cerebellum after prenatal and postnatal Pb exposure. Pregnant female rats were randomly divided into three groups: control, Pb (0.3% Pb acetate in drinking water), and Pb plus gintonin (100 mg/kg, p.o.). Blood Pb was increased in dams and pups; gintonin treatment significantly decreased blood Pb. On postnatal day 21, the number of degenerating Purkinje cells was remarkably increased while the number of calbindin-, GAD67-, NMDAR1-, LPAR1-immunoreactive intact Purkinje cells, and GABA transporter 1-immunoreactive pinceau structures were significantly reduced in Pb-exposed offspring. Following Pb exposure, gintonin ameliorated cerebellar degenerative effects, restored increased pro-apoptotic Bax, and decreased anti-apoptotic Bcl2. Gintonin treatment attenuated Pb-induced accumulation of oxidative stress (Nrf2 and Mn-SOD) and inflammation (IL-1β and TNFα,), restoring the decreased cerebellar BDNF and Sirt1. Gintonin ameliorated Pb-induced impairment of myelin basic protein-immunoreactive myelinated fibers of Purkinje cells. Gintonin attenuated Pb-induced locomotor dysfunctions. The present study revealed the ameliorating effects of gintonin against Pb, suggesting the potential use of gintonin as a preventive agent in Pb poisoning during pregnancy and lactation.

## 1. Introduction

Ginseng ***(Panax ginseng*** C.A. Meyer), a member of the Araliaceae plant family, has been used as a tonic to improve health and body functions. Ginseng has been cultivated for thousands of years in many Asian countries. Its roots have been used as one of important ingredients in traditional herb medicines. Especially, the ginseng extract exhibits neuroprotective effects in neurodegenerative diseases [1,2,3,4,5]. Until now, ginsenosides, the saponin component from ginseng, have been regarded as biologically active component and investigated as the target of research [5,6]. However, other active ingredients of ginseng excluding ginsenosides remain unknown. Recently, a novel G protein-coupled lysophosphatidic acid receptor (LPAR) ligand, designated gintonin, has been isolated from ginseng. Gintonin, a non-acidic polysaccharide and a non-saponin, targets LPA receptors (LPARs) in neuronal and non-neuronal cells [7]. Gintonin-mediated LPAR activation is coupled to [Ca^2+^]_i_ transients in neuronal and non-neuronal cells [8] and is further linked to in vitro calcium-dependent intra- and inter-cellular communications through the regulation of ion channels and receptors [8]. Oral administration of gintonin has shown diverse beneficial effects, including anti-metastatic effects, anti-dementia effects, enhancing brain neurogenesis and plasticity, and improving learning and memory functions [1,9].

Brain development is intricately regulated by complexly orchestrated molecular mechanisms. The early postnatal period in rodents, which is equivalent to the late third trimester gestational period in humans, is critical for brain development [10]. Especially, during the early postnatal period in rodents, maximum neurogenesis, subsequent migration, synaptogenesis, and maturation occur in the cerebellum and hippocampus and the volume of the brain increases significantly [10,11]. It is well-known that LPAs are developmentally bioactive molecules, promoting cell proliferation, migration, differentiation, myelination, and synapse formations in the brain [12]. In addition, LPA1-deficient mice exhibit brain maldevelopment and brain dysfunctions in cognition and emotion [13]. Thus, the LPA-LPAR1 ***axis*** plays an important role in the mouse brain development, although the LPAR1 expression is gradually diminished after the postnatal stage [14].

Among several regions of the brain, the cerebellum is important for fine control of movement, motor learning and cognition, maintaining balance, and motor coordination. The cerebellum is developmentally immature in humans, dogs, cats, and rodents relative to cattle and horses that show almost complete cerebellar development at birth [15]. Rodent brain development starts during the gestational period and continues in the postnatal period. Especially, the early postnatal period is critical for the normal development and maturation of the cerebellum. During this critical window, the developing brain is susceptible to exogenous materials such as chemicals, heavy metals (i.e., lead), and toxicants that alter regulating molecular signal cascades [16,17,18,19].

In the list of hazardous waste priority from the US Environmental Protection Agency, lead (Pb) is ranked second after arsenic. Pb is environmentally prevalent owing to its wide use in lead-based batteries, architectural lead sheets, silver smelting, water pipelines, adulterating wine, sheathing material, solder for electronics, leaded gasoline, and paints [20]. Notably, environmental Pb pollution in China and USA is a serious health problem owing to the massive production and consumption of Pb in these countries [20,21]. Additionally, the occupational and environmental exposure to Pb remains a health-threatening problem in many countries including Korea [21,22]. Ingested or inhaled Pb can easily cross the blood–brain barrier. In the developing brain, multiple areas including the amygdala, cerebellum, and hippocampus are primary targets for Pb accumulation [23]. However, relatively is little known whether herbal medicine(s) such as ginseng could afford brain protection against Pb poisoning. Although previous studies have shown that gintonin exhibits neuroprotective effects in the brain against oxidative stress and inflammations [1,3,5], it is not specified whether gintonin, which abundantly contains LPAs, can attenuate Pb-induced neurotoxicity in the developing rat cerebellum. In the present study, we investigated whether gintonin treatment following pre- and post-natal Pb exposure could circumvent structural and functional deterioration in the developing cerebellum. Our results showed that gintonin treatment attenuated Pb-induced cerebellar maldevelopments and cerebellum-mediated malfunctions. We further discussed the molecular mechanisms by which gintonin attenuates Pb-induced cerebellar neurotoxicity, indicating the possibility that gintonin can be a useful agent to prevent Pb poisoning during pregnancy.

## 2. Materials and Methods

### 2.1. Experimental Design and Animals

Female (n = 15) and male (n = 5) Sprague–Dawley rats (7 weeks old) were purchased from Narabiotec Co., Ltd. (Seoul, Republic of Korea) and housed under conditions of adequate temperature (23 °C) and humidity (60%), with a 12 h light/12 h dark cycle. The animals were acclimatized for 1 week to a conventional environment at the animal facility at the College of Veterinary Medicine. Animals were allowed free access to food (Purina LabDiet 5008, Purina Korea, Seongnam, Korea) and tap water. The present experimental procedures were approved by the Institutional Animal Care and Use Committee of the Konkuk University (Permit No: KU18133) and followed the guidelines of the ‘National Institutes of Health Guide for the Care and Use of Laboratory Animals, issued by the Institute of Laboratory Animal Resources, USA, 1996. Animals were handled and cared to reduce stress caused by the employed procedures.

### 2.2. Preparation of Gintonin

Briefly, 1 kg of 4-year-old ginseng (Korea Ginseng Corporation, Daejeon, Republic of Korea) was processed to extract gintonin using methods previously described [2,8]. Gintonin consists of carbohydrates, lipids, ginseng proteins, and other minor components; approximately 30% carbohydrates, 20.2% lipids, and 30.3% proteins. The lipid compositions of gintonin were analyzed by LC-MS/MS: fatty acids (7.53% linoleic acid, 2.82% palmitic acid, and 1.46% oleic acid), 0.60% lysophospholipids and phospholipids, and 1.75% phosphatidic acids. The total lipid content in gintonin is approximately 14.2% [24].

### 2.3. Administration of Lead Acetate and Gintonin

**Figure 1:**
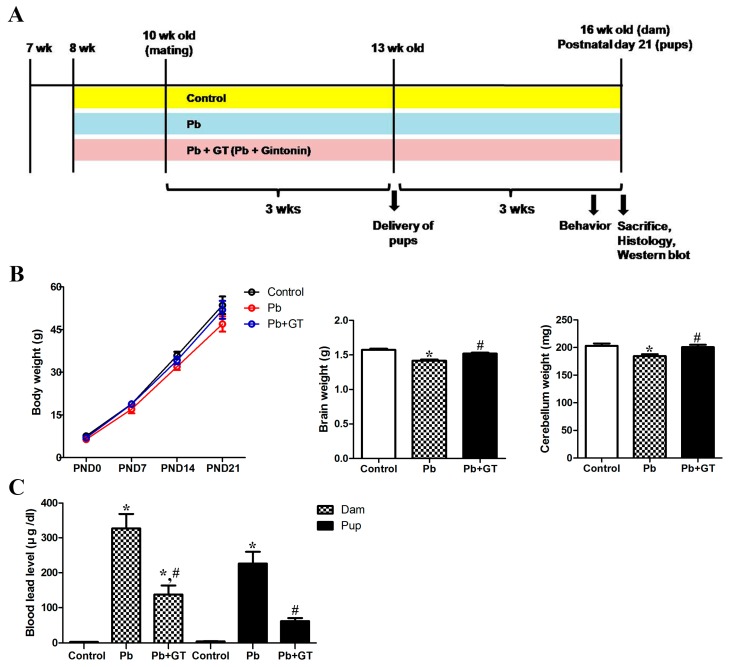
Experimental design and physiological changes after Pb alone or gintonin plus Pb. (**A**) The present study was performed as shown in the experimental design. (**B**) Body weights of dams (n = 3 per group) during gestation and offspring (n = 12 per group) on postnatal days (PND) 0, 7, 14, and 21 in the control, Pb, and Pb plus gintonin (Pb + GT) groups (body weight, *p* = 0.9676, one-way analysis of variance). Whole-brain and cerebellar weights of offspring on PND21 (wet brain weight, *p* < 0.0001, one-way analysis of variance; wet cerebellar weight, *p* = 0.0080, one-way analysis of variance). (**C**) Blood Pb levels in the dams and offspring PND21 (blood Pb level, *p* < 0.0001, one-way analysis of variance). * *p* < 0.05, compared to control group, # *p* < 0.05, compared to Pb alone, Tukey’s post-hoc test. The bars indicate means ± standard error of mean.

Adult female Sprague–Dawley rats were randomly divided into three groups: control group (n = 5), Pb group (n = 5), and Pb plus gintonin (Pb + GT) group (n = 5). The design of the experiment (Figure 1) and the doses of gintonin and Pb administered were adopted from previous studies [9,25,26]. Pb acetate (0.3%; Sigma-Aldrich, USA) was dissolved in distilled water with glacial acetic acid (0.05%; Junsei Chemical Co., Tokyo, Japan) to prevent Pb precipitation. Gintonin (100 mg/kg), which was orally administered, was freshly prepared in saline daily. To account for the effects of stress during oral intubation, rats in the control and Pb groups were orally administered the same volume of saline. Before mating, female rats received Pb in the drinking water and gintonin via oral intubation for 2 weeks. Pregnancy was confirmed when sperm were detected on vaginal smears or when the presence of vaginal plugs was confirmed. The day of pregnancy confirmation was designated as day 0. Male and female rats were separated and pregnant females were singly housed in each cage for safe delivery and caring of offspring until the end of the experiments. Pb and gintonin administration continued during gestation and delivery of offspring, until the end of the experiment on postnatal day (PND) 21. The body weights of the offspring were recorded and averaged every week. To preclude any effect of litter size among the different experimental groups, six rat pups per cage (total 30 pups per group) were selected for the experiment and the remaining offspring were killed. Whenever possible, only male offspring were used for the experiment. Female offspring were used only if necessary, to maintain equal litter sizes. Before killing, the sex of every offspring was recorded. The researchers in the present study performed all experimental procedures carefully to minimize suffering and the number of animals used.

### 2.4. Locomotor Coordination Assay (Bar Holding Test and Wire Mesh Ascending Test) in Offspring

From the 2nd to 3rd postnatal week, eye-opening and increased movements enhance the cerebellar development and motor control functions [27]. Therefore, we assessed the motor coordination of PND19 offspring using two methods described in our previous study [26]. In the bar holding test, offspring were allowed to grasp a stainless-steel bar (0.7 cm in diameter and 35 cm long) that was suspended 30 cm above a soft padded surface. The time spent grasping the bar with the forelimbs was measured for a maximum of 60 s. During the wire mesh ascending test, a 0.7 cm thick stainless-steel mesh (45 cm long × 30 cm wide) was placed at an angle of 26° in a water bath containing water at 25 °C, such that the wire mesh was 30 cm above the water surface. The offspring was placed with its quarter hind and tail dipped in the water. After three training trials, the time taken to reach the top of the mesh was recorded over a 30 s period.

### 2.5. Analysis of Pb Level in the Blood Using Atomic Absorption Spectrometry and Measurement of Cerebellar Weight

On PND21, five dams per group and PND21 offspring (n = 15 per group) were anesthetized with an intraperitoneal injection of 1.5 g/kg urethane (Sigma-Aldrich). Blood samples for analysis were collected from dams and offspring by cardiac puncture. As described in the previous study [28], procedures for blood Pb analyses were performed in the Neodin Biovet Laboratory (Seoul, Korea), certified by the Korean Ministry of Health and Welfare. Blood Pb levels were analyzed using the PinAAcle 500 flame atomic absorption spectrometer (Perkin Elmer Zeeman 5100; Norwalk, CT, USA) and an HGA-600 graphite furnace with Zeeman background correction. In terms of external quality control, the Neodin has passed the German External Quality Assessment Scheme operated by the Friedrick Alexander University. The spiked Pb sample demonstrated good percentage recoveries, and the limit of detection was 0.070 μg/dL. The absorption wavelength was 283.3 nm, and the r^2^ of the calibration curve exceeded 0.995. Additionally, the brains were dissected and wet weighed. The cerebella were immediately dissected, wet weighed, and frozen until use.

### 2.6. Tissue Processing, Nissl Staining, and Immunohistochemistry

For histology, the remaining PND21 offspring (n = 15 per group) were also anesthetized with urethane and perfused transcardially with phosphate-buffered saline (0.1 M, pH 7.4), followed by 4% paraformaldehyde in 0.1 M phosphate buffer (pH 7.4). The cerebella were removed and post-fixed in the same fixative overnight at 4 °C. Cerebellar vermis was sagittally embedded in paraffin blocks, and 5-μm-thick sections were obtained using a microtome (Leica, Germany). Three sections per offspring were used, and a total of 36 paraffin sections per group were analyzed. Nissl staining was performed using routine procedures. Immunohistochemistry for target marker proteins was subsequently performed as described in our previous study [29]. Briefly, deparaffinized sections were subjected to antigen retrieval, quenching with 0.3% hydrogen peroxide (H_2_O_2_), and blocking with 10% normal horse serum. Then, the sections were incubated with primary antibodies overnight at 4 °C: calbindin-28kD (CB, 1:5000; Swant, Bellinzona, Switzerland), Bax (rabbit, 1:200, GeneTex, Irvine, CA, USA), LPAR1 (rabbit, 1:1000, Abcam, Cambridge, UK), ***N***-methyl-_D_-aspartate receptor 1 (NMDAR1, rabbit, 1:1000, Millipore, Billerica, MA, USA), NMDAR2B (mouse, 1:500, BD Bioscience, San Jose, CA, USA), glutamate decarboxylase (GAD) 67 (1:1000, Stressgen, Victoria, BC, Canada), γ-aminobutyric acid (GABA) transporter 1 (GABAT1, 1:500; Synaptic Systems, Göttingen, Germany), myelin basic protein (MBP; rabbit, 1:3000, Millipore), or oligodendrocyte transcription factor 2 (Olig2, 1:500; R&D systems, Minneapolis, MN, USA). Subsequently, the sections were exposed to biotinylated immunoglobulin G (1:200; Vector, Burlingame, CA, USA) and streptavidin–peroxidase complex (1:200; Vector). Finally, they were visualized using 3,3′-diaminobenzidine tetrachloride (Sigma-Aldrich). Additionally, hematoxylin counter-staining was performed for some sections. The sections were finally dehydrated, cleared, and cover-slipped in a toluene-based mounting medium (Richard-Allan Scientific, Thermo Scientific, MI, USA).

Histological analyses were performed by an investigator blinded to rat treatments. The method of quantification employed in the present study was conducted as previously described [25,26]. The pyknotic, degenerating, Nissl-stained Purkinje cells and marker protein-immunostained intact Purkinje cells, with a demarcated nucleus and cytoplasm, were counted. The observations were carried out in the 2nd, 5th, 7th, and 8th lobules of the sagittal sections of the cerebellar vermis [25].

### 2.7. Western Blotting

Immunoblotting for target marker proteins was performed as described in our previous study [29]. To detect Bax, Bcl2, cleaved caspase3, synaptophysin, NMDAR1, GAD67, LPAR1, brain-derived neurotrophic factor (BDNF), Nrf2, sirtuin 1 (Sirt1), manganese superoxide dismutase (Mn-SOD), MBP, interleukin 1β (IL1β), and tumor necrosis factor α (TNFα) protein expression in the cerebellum of PND21 pups (n = 15 per group), the frozen samples were homogenized in RIPA lysis buffer, centrifuged at 15,000× ***g***, and the supernatant was separated. Proteins were quantified using the Thermo Pierce^®^ BCA protein assay kit (Thermo Fisher Scientific, Rockford, IL, USA). Aliquots containing 40 μg of total protein were boiled in the loading buffer containing 150 mM Tris (pH 6.8), 3 mM dithiothreitol, 6% sodium dodecyl sulfate (SDS), 0.3% bromophenol blue, and 30% glycerol. The aliquots were then loaded onto a 10% SDS-polyacrylamide gel, and the proteins were separated. After electrophoresis, the gels were transferred to polyvinylidene fluoride membranes (Roche, Penzberg, Germany). The membranes were blocked by incubation in 5% skimmed milk in Tris-buffered saline (TBS; pH 7.4) for 1 h. Next, they were incubated overnight at 4 °C with the primary antibody against Bax (rabbit, 1:2000, GeneTex), Bcl2 (rabbit, 1:2000, GeneTex), cleaved caspase3 (rabbit, 1:2000, Abcam), synaptophysin (rabbit, 1:5000, Abcam), NMDAR1 (rabbit, 1:1000, Millipore), GAD67 (1:1000, Stressgen), LPAR1 (rabbit, 1:1000, Abcam), BDNF (rabbit, 1:1000, Novus, Littleton, CO, USA), Nrf2 (rabbit, 1:1000, Abcam), Sirt1 (mouse, 1:1000; Santa Cruz Biotechnology, CA, USA), Mn-SOD (SOD2, goat, 1:1000, Santa Cruz), MBP (rabbit, 1:2000; Abcam), IL1β (rabbit, 1:1000, GeneTex), or TNFα (rabbit, 1:2000, Abcam). The blots were washed three times in TBS containing 0.1% Tween-20 and then incubated with a horseradish peroxidase-conjugated secondary antibody (1:2000). Bands were visualized using SuperSignal^®^ West Pico Chemiluminescent Substrate (Thermo Fisher Scientific, Rockford, IL, USA). After several rounds of blotting, the relative optical density of each band was measured using NIH ImageJ software.

### 2.8. Statistical Analysis

All data are expressed as means ± standard errors of the mean for each group, and the significance of the differences between these mean values was determined using one-way analysis of variance, followed by Tukey’s post-hoc test for multiple comparisons. All analyses were performed using GraphPad Prism ver. 5.01 (GraphPad Software, Inc.; La Jolla, CA, USA). Significance was denoted at *p* < 0.05.

## 3. Results

### 3.1. Effects of Gintonin Administration on Pb-Induced Changes in Body Weight, Cerebellar Weight, and Blood Pb Levels

First, according to the experimental design, we investigated the changes in physiological parameters in the dams and offspring on PND21 (Figure 1A). The body, brain, and cerebellar weights were slightly decreased by long-term Pb exposure, with gintonin recovering these weight losses induced by Pb exposure in the offspring (Figure 1B). Prenatal and postnatal Pb exposure significantly increased the blood Pb level in both dams and offspring (Figure 1C). Since the ingested Pb can move from the dam to offspring via the placenta and milk [20], the Pb-exposed dam demonstrated higher blood Pb levels than the offspring (Figure 1C). However, gintonin significantly decreased the Pb levels in both dams and offspring (Figure 1C). Thus, gintonin treatment could attenuate the transfer of Pb from mothers to offspring. These results indicate that gintonin treatment could increase the excretion of blood Pb from both dams and offspring.

### 3.2. Gintonin Treatment Attenuates Pb-Induced Damages in the Developing Cerebellum

**Figure 2:**
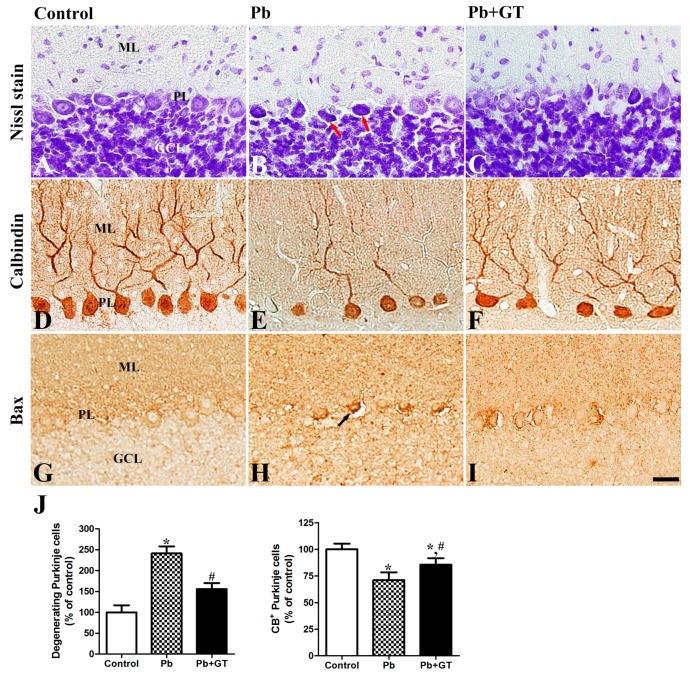
Effects of gintonin on Pb-induced cerebellar Purkinje cell impairments. Nissl staining (**A**–**C**), immunohistochemistry for calbindin (CB) (**D**–**F**), and immunohistochemistry for Bax (**G**–**I**) in the cerebellum of offspring from control, Pb, and Pb plus gintonin (Pb + GT) groups. GCL, granular cell layer; ML, molecular layer; PL, Purkinje cell layer. Bar = 25 μm. Degenerating Purkinje cells (red arrow), CB-immunoreactive Purkinje cells, bax-immunoreactive cells (arrow) are detected in the Purkinje cell layer. (**J**) The numbers of degenerating Purkinje cells (*p* < 0.0001, one-way analysis of variance) and CB-immunoreactive Purkinje cells (*p* < 0.0001, one-way analysis of variance) in the cerebellum are expressed as percentages of the value in the control group (n = 12 per group). * *p* < 0.05, compared to control group; # *p* < 0.05, compared to Pb alone, Tukey’s post-hoc test. The bars indicate means ± standard error of the mean.

Based on the abilities of gintonin to influence neurogenesis and neuroprotection in the brain and brain functions [3,5,9] and to reduce blood Pb amount (Figure 1C), we examined the cerebellar protective effects of gintonin in the Pb-intoxicated offspring. Nissl staining demonstrated the formation of three layers in the cerebellar cortex, namely, the molecular layer (ML), Purkinje cell layer (PL), and granular cell layers (GCL), which were normally developed in all groups. However, distinct differences were observed among groups, i.e., the number of intact Purkinje cells was lower in the Pb alone-exposure than in the control (Figure 2A,B) but gintonin treatment partially restored the Pb-induced reduction in intact Purkinje cells (Figure 2C,J). The Pb-induced increase in degenerating pyknotic Purkinje cells was also reduced by gintonin treatment (Figure 2B, red arrows). Since calbindin 28kD is a specific marker of Purkinje cells [30], we examined and detected CB immunoreactivity in the cell bodies and dendrites of Purkinje cells. The number of CB-immunoreactive Purkinje cells was lower in the Pb alone-exposure compared to the control (Figure 2D,E); gintonin treatment partially restored CB-immunoreactive Purkinje cells (Figure 2F). Especially, dendritic branching development of CB-immunoreactive Purkinje cells were prominently impaired owing to Pb exposure relative to the control. However, co-administration of gintonin with Pb restored dendritic branching development (Figure 2D–F). Gintonin alone (100 mg/kg) treatment did not affect cerebellar Purkinje cells (data not shown). Present findings suggest that gintonin may attenuate Pb-induced cerebellar impairments. These results also demonstrated that long-term Pb exposure in dams impairs the developmental Purkinje cell integrity in the offspring. Gintonin treatment attenuates Pb-induced cerebellar Purkinje cell impairments.

### 3.3. Gintonin Treatment Attenuates Pb-Induced Changes in BDNF, Sirt1, and Apoptosis-Related Bax and Bcl2 Protein Expression

**Figure 3:**
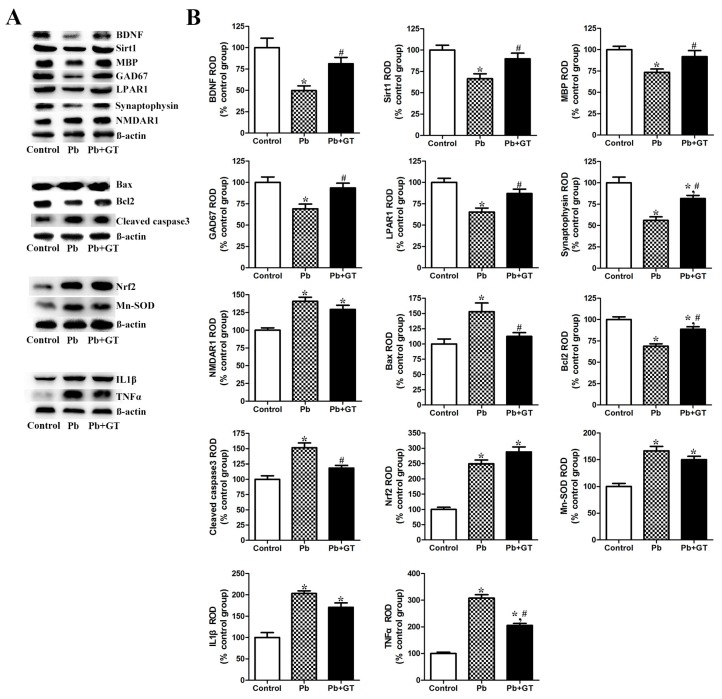
Effects of gintonin on Pb-induced cerebellar changes in various protein expression levels. Representative immunoblots for BDNF, Sirt1, MBP, GAD67, LPAR1, synaptophysin, NMDAR1, Bax, Bcl2, cleaved-caspase3, Nrf2, Mn-SOD, IL-1β, and TNFα (**A**,**B**) in the cerebellum of pups from control, Pb, and Pb plus gintonin (Pb + GT) groups at postnatal day 21 (PND21). Relative optical density (ROD) of each of immunoblot bands for BDNF (*p* = 0.0010, one-way analysis of variance), Sirt1 (*p* = 0.0020, one-way analysis of variance), MBP (*p* < 0.0001, one-way analysis of variance), GAD67 (*p* = 0.0022, one-way analysis of variance), LPAR1 (P = 0.0001, one-way analysis of variance), synaptophysin (*p* < 0.0001, one-way analysis of variance), NMDAR1 (*p* < 0.0001, one-way analysis of variance), Bax (*p* = 0.0018, one-way analysis of variance), Bcl2 (*p* < 0.0001, one-way analysis of variance), cleaved-caspase3 (*p* < 0.0001, one-way analysis of variance), Nrf2 (*p* < 0.0001, one-way analysis of variance), Mn-SOD (*p* < 0.0001, one-way analysis of variance), IL-1β (*p* < 0.0001, one-way analysis of variance), and TNFα (*p* < 0.0001, one-way analysis of variance) is demonstrated as a percentage of the value in the control group (n = 12 per group). * *p* < 0.05, compared to control group; # *p* < 0.05, compared to Pb alone, Tukey’s post-hoc test. The bars indicate the means ± standard error of the mean.

BDNF is an important neurotrophic factor in various brain developmental processes, including cellular proliferation/survival, differentiation, branching of the dendritic arbor, spine formation, and synaptic formation [29]. Sirt1 is an epigenetic factor that is involved in several important biological processes, including synaptic plasticity, neuroprotection, learning, and memory via the regulation of cAMP-response element binding protein (CREB) and BDNF expression in the brain [29,31]. BDNF and Sirt1 protein expression levels were reduced with Pb administration, while gintonin treatment restored the reduced BDNF and Sirt1 protein levels (Figure 3).

Owing to the increase in the number of degenerating cells such as Purkinje cells in the cerebellum of the Pb-exposed offspring, we compared the expression patterns of the proapoptotic Bax protein in the cerebellar cortex by using immunohistochemistry and immunoblotting assays. Bax-immunoreactivity was not detected in the control group (Figure 2G), while Bax expression was increased following long-term Pb exposure in the ML, PL, and GCL of the cerebellum (Figure 2H). However, gintonin administration decreased the Pb-induced increase in Bax in the three cerebellar layers (Figure 2I). Using immunoblotting, we confirmed a similar pattern of altered expression in proapoptotic proteins, including Bax and cleaved caspase3 (activated form of caspase3) in the whole cerebellum; gintonin reduced the Pb exposure-induced increase in Bax and cleaved caspase3 expression (Figure 3A,B). However, anti-apoptotic Bcl2 expression was diminished by Pb; gintonin treatment restored the Pb-induced reduction in cerebellar Bcl2 expression (Figure 3). These results indicate that Pb increases proapoptotic protein expression, whereas gintonin treatment increases anti-apoptosis-related protein expression.

### 3.4. Gintonin Treatment Attenuates Pb-Induced Changes in LPAR1, Presynaptic Synaptophysin, and Post-NMDAR1 Expression

**Figure 4:**
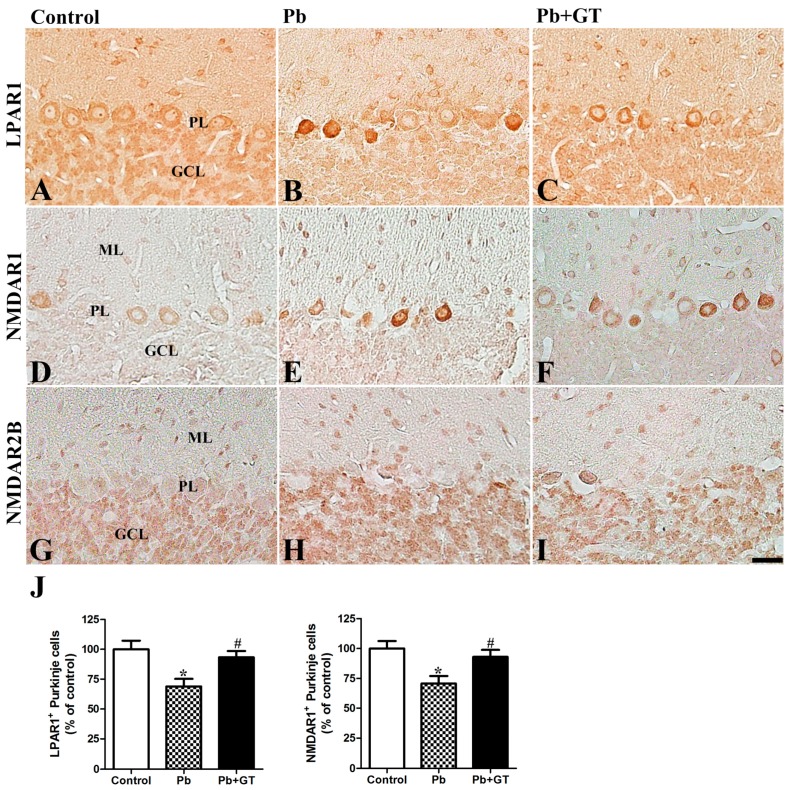
Effects of gintonin on Pb-induced changes in cerebellar LPAR1 and NMDA receptor expression. Immunohistochemistry of LPAR1 (**A**–**C**), NMDAR1 (**D**–**F**), and NMDAR2B (**G**–**I**) in the cerebellum of offspring from control, Pb, and Pb plus gintonin (Pb + GT) groups. LPAR1 and NMDAR1 are mainly detected in the Purkinje cell layer, and NMDAR2B is detected in the granular cell layer in the cerebellar cortex. The number of LPAR1-immunoreactive and NMDAR1-immunoreactive intact Purkinje cells is significantly reduced, while gintonin treatment ameliorated this reduction in the cerebellum. GCL, granular cell layer; ML, molecular layer; PL, Purkinje cell layer. Bar = 25 μm. (**J**) Numbers of LPAR1-immunoreactive Purkinje cells (*p* = 0.0031, one-way analysis of variance) and NMDAR1-immunoreactive Purkinje cells (*p* = 0.0042, one-way analysis of variance) are expressed as a percentage of the value in the control group in the cerebellar cortex (n = 12 per group). * *p* < 0.05, compared to control group; # *p* < 0.05, compared to Pb alone, Tukey’s post-hoc test. The bars indicate means ± standard error of the mean.

LPA and LPARs play key roles in the developing brain as LPA1-deficient mice show abnormal brain development [32]. However, the expression pattern of LPARs in Pb-exposed rats has not been clearly defined. In the cerebellum of PND21 rats, LPAR1, the main subtype of LPARs in the brain, was detected in the PL of the cerebellar cortex. Pb exposure reduced the number of LPAR1-immunoreactive Purkinje cells, while gintonin treatment demonstrated protective effects against the Pb-induced decrease in LPAR1 expression in these cerebellar cells (Figure 4). Furthermore, we examined the changes in LPAR1 expression levels after Pb exposure. Although the LPAR1 level decreased after Pb exposure, gintonin treatment restored the LPAR1 expression level (Figure 3).

We investigated NMDAR1 and NMDAR2B post-synaptic expression in the cerebellum since glutamatergic excitotoxicity is an important mechanism of Pb-induced neurotoxicity [33]. NMDAR1 was mainly detected in the PL and some cells in the ML, while NMDAR2B was immunostained in the GCL in the cerebellar cortex. NMDAR1 is an essential subtype of the NMDA receptor complex and NMDAR2B endows functional characteristics and therapeutic targets for the treatment of neurological diseases [11]. Owing to Pb exposure, the number of NMDAR1-positive Purkinje cells was significantly reduced; gintonin treatment ameliorated the Pb-induced decrease in NMDAR1 (Figure 4). Interestingly, we observed different results between the immunostaining intensity of NMDAR1 and immunoblot assay of NMDAR1, with the immunoblot assay demonstrating an increase rather than a decrease in NMDAR1 expression (Figure 3). These results suggest the possibility that although NMDAR1 was reduced after Pb exposure in individual Purkinje cells, NMDAR1 expression levels in other cells in the cerebellar cortex might be compensatively increased in the whole cerebellum. Contrary to the NMDAR1 expression in the Purkinje cells, NMDAR2B was detected in the GCL, and the change induced by Pb and gintonin was insignificant (Figure 4).

Synaptophysin is mainly expressed in presynaptic vesicular membranes and is a useful marker of synaptic development in the brain [18,29]. In our previous study, synaptophysin was immunohistochemically detected in the ML and GCL in the cerebellar cortex [18]. The present immunoblot assay demonstrated that the expression of synaptophysin was lower in the Pb-alone exposure group than in the control; gintonin treatment restored these levels in the cerebellum (Figure 3). These results suggested that Pb exposure impairs presynaptic and post-synaptic protein expression, and gintonin treatment restored the pre- and post-synaptic damages at the molecular level.

### 3.5. Gintonin Treatment Attenuates Pb-Induced Changes in GAD and GABAT1

**Figure 5:**
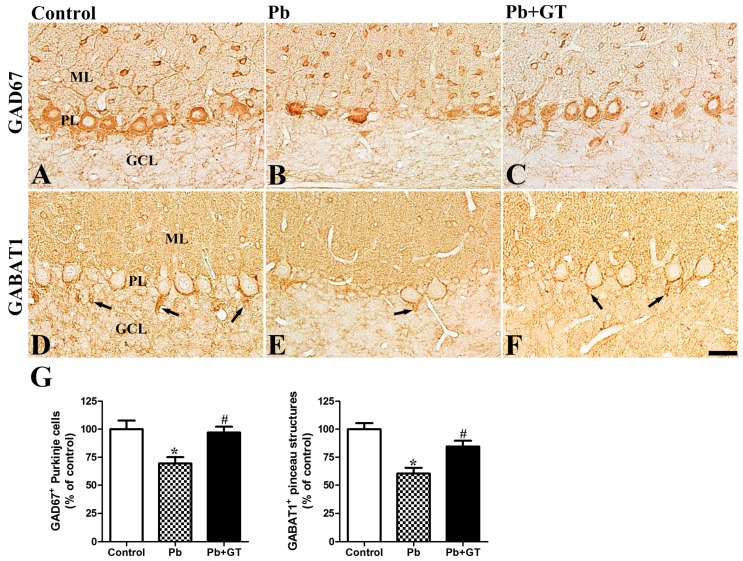
Effects of gintonin on Pb-induced changes in cerebellar GAD67 and GABA transporter1. Immunohistochemistry for GAD67 (**A**–**C**) and GABA transporter 1 (GABAT1) (**D**–**F**) in the cerebellum of offspring from control, lead (Pb), and Pb plus gintonin (Pb + GT) groups. Note that GAD67-immunoreactive Purkinje cells and GABAT1-positive pinceau structures (arrow) near Purkinje cells are detected in the cerebellar cortex and are significantly reduced in the Pb group; gintonin treatment attenuates these reductions in the Pb + GT group. GCL, granular cell layer; ML, molecular layer; PL, Purkinje cell layer. Bar = 25 μm. (**G**) The number of GAD67-positive Purkinje cells (*p* = 0.0017, one-way analysis of variance) and GABAT1-positive pinceau structures (*p* < 0.0001, one-way analysis of variance) is expressed as a percentage of the value in the control group in the cerebellar cortex (n = 12 per group). * *p* < 0.05, compared to control group; # *p* < 0.05, compared to Pb alone, Tukey’s post-hoc test. The bars indicate means ± standard error of mean.

GABA is an important regulator in the morphogenesis of the developing brain [34]. In all groups, GABA-synthesizing GAD67 was detected mainly in the PL. In addition to the GABAergic Purkinje cells, GAD67-immunoreactive cells were also observed in the ML, where the GABAergic interneurons such as basket cells and stellate cells reside. Pb exposure significantly reduced the number of GAD67-immunoreactive Purkinje cells, and GAD67-immunoreactive dendritic branching of the Purkinje cells was also impaired relative to the control group. Gintonin treatment acted against Pb-induced neurotoxicity by protecting the development of GAD67-immunoreactive Purkinje cells in the cerebellar cortex (Figure 5). Immunoblot analysis showed a similar pattern of GAD67 expression with that of GAD67-immunoreactive Purkinje cells in the whole cerebellum (Figure 3). Additionally, GABAT1, a mediator of GABA uptake, was detected in pinceau structures enclosing the Purkinje cell somata and scattered puncta in the ML. Developmentally, GABAT1 is expressed in the pinceau structure as GABAergic synapses begin to form, and GABAT1 mediates GABA uptake from the synaptic cleft [27]. Similar to the Pb-induced change in Purkinje cells, the GABAT1-immunoreactive pinceau structures were also reduced after Pb exposure. However, gintonin treatment ameliorated the Pb-induced reduction of GABAT1 immunoreactive pinceau structures in the cerebellar cortex (Figure 5). Therefore, these results indicated that although the synapses (pinceaus) between the basket cells and Purkinje cells were impaired following Pb exposure, gintonin treatment protected GABA-related synapses from the Pb-induced damages (Figure 5).

### 3.6. Effects of Gintonin Treatment on Pb-Induced Changes in Nrf2, Mn-SOD, IL-1β, and TNFα Expression

Next, we investigated the effect of gintonin on Pb-induced changes in oxidative stress-related markers. Nrf2 was significantly increased following Pb exposure and gintonin treatment further increased the expression of Nrf2 in the whole cerebellum. Additionally, we observed that the antioxidant Mn-SOD was significantly increased in the cerebellum following Pb exposure and gintonin treatment slightly reduced the cerebellar expression level of Mn-SOD (Figure 3). These results indicate that gintonin differentially regulates oxidative stress-related marker proteins after Pb exposure. Additionally, we focused on the inflammatory mediators IL-1β and TNFα in the developing cerebellum. Both IL-1β and TNFα are significantly higher in the cerebellum of the Pb-alone group than in the control group. However, gintonin treatment abrogated the Pb-induced upregulation of inflammatory cytokines (Figure 3). These results suggested that Pb exposure induces neuroinflammation in the cerebellum, while gintonin demonstrated anti-inflammatory effects.

### 3.7. Effects of Gintonin Co-Administration with Pb on MBP, Olig2, and Locomotive Functions in the Developing Cerebellum

Along with the changes in the somata and dendritic arbor of Purkinje cells, we investigated the expression of MBP, which is one of the main protein components in the myelin sheath of nerve fibers, required for coordinated motor regulations in the cerebellum [35]. We investigated the cerebellum of PND21 rats when the three layers had developed to a similar extent as in the adult in situ. MBP was detected in the myelinated axons from Purkinje cells of gray matter and myelinated fiber tracts of white matter in the cerebellum, and MBP immunoreactivity decreased following Pb exposure. However, gintonin treatment ameliorated the Pb-induced MBP immunoreactivity reduction. The protein expression pattern of MBP coincides with the MBP immunoreactivity in the cerebellum; gintonin treatment restored the Pb-induced reduction in MBP protein level, as shown in the immunoblotting assay (Figure 3).

**Figure 6:**
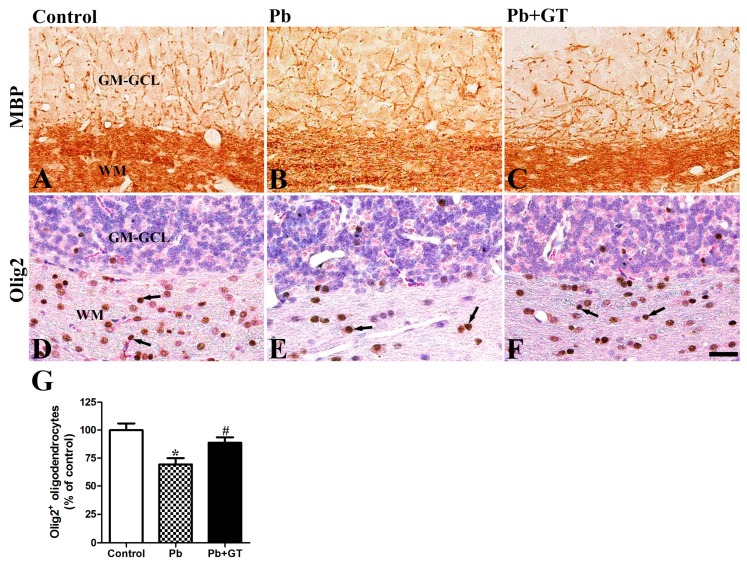
Effects of gintonin on Pb-induced cerebellar MBP and Olig2. Immunohistochemistry of MBP (**A**–**C**) and Olig2 (**D**–**F**) in the cerebellum of offspring from control, Pb, and Pb plus gintonin (Pb + GT) groups. Note that MBP-positive myelinated fibers and Olig2-immunoreactive oligodendrocytes (arrow) are detected in the cerebellum. Pb-induced reduction in the number of Olig2-immunoreactive oligodendrocytes is significantly ameliorated by gintonin treatment. The intensity of MBP staining in white matter (WM) (**B**) decreases after Pb exposure, with gintonin treatment ameliorating these observed reductions. MBP, Myelin basic protein; GM-GCL, granular cell layer in gray matter; WM, white matter. Bar = 25 μm. (**G**) The number of Olig2-immunoreactive oligodendrocytes (*p* = 0.0011, one-way analysis of variance) in the cerebellar cortex is expressed as percentages of the value in the control group (n = 12 per group). * *p* < 0.05, compared to control group; # *p* < 0.05, compared to Pb alone, Tukey’s post-hoc test. The bars indicate the means ± standard error of the mean.

Additionally, myelin sheath-forming Olig2-immunoreactive oligodendrocytes were observed in all areas of the cerebellum, with most located at the cerebellar white matter to wrap axons. In the cerebellum of the Pb-exposed offspring, the number of Olig2-immunoreactive oligodendrocytes was significantly reduced compared to the control groups. However, gintonin treatment protected the cerebellum from Pb-induced impairment by sparing the number of Olig2-immunoreactive oligodendrocytes (Figure 6, arrows in WM).

**Figure 7:**
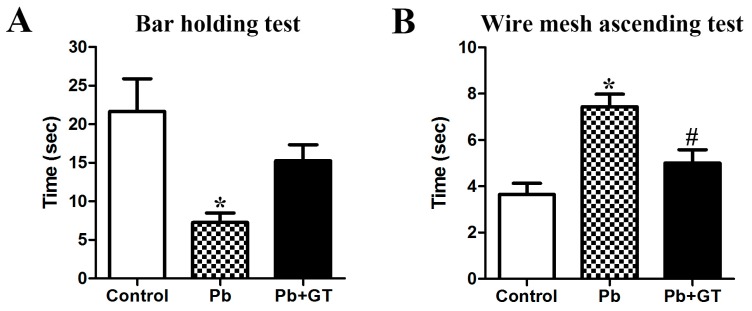
Effects of gintonin on Pb-induced locomotive impairments. Effect of Pb exposure and gintonin treatment on the bar holding test (**A**) and wire mesh ascending test (**B**) among offspring from control, Pb, and Pb plus gintonin (Pb + GT) groups. (**A**) The time during which the animal stayed on the bar (*p* < 0.0001, one-way analysis of variance) in the bar holding test. (**B**) The time spent to reach the top of the wire mesh (*p* = 0.0082, one-way analysis of variance) in the wire mesh ascending test (n = 12 per group). * *p* < 0.05, compared to control group; # *p* < 0.05, compared to Pb alone, Tukey’s post-hoc test. The bars indicate the means ± standard error of the mean.

We next examined whether the structural deficit by Pb exposure was related to cerebellar motor dysfunctions. Hence, we examined two kinds of locomotive tests, the bar holding and wire mesh ascending tests. In the bar holding test, the time spent grasping the bar was significantly lower in the offspring of Pb-exposed dams than in the control group. Gintonin treatment increased time spent grasping the bar with no statistical significance (Figure 7A). In the ascending wire mesh test, the time spent ascending the wire mesh was delayed in the Pb-exposed pups; gintonin administration significantly shortened the time required to reach the top (Figure 7B). These results indicate that long-term Pb exposure in dams induces motor dysfunctions in offspring and gintonin treatment to dams ameliorates Pb-induced motor dysfunctions in the offspring.

## 4. Discussion

We have previously shown that gintonin is a novel ginseng-derived glycolipoprotein complex. Its main functional ingredients are LPAs [7]. Additionally, we have reported that gintonin, via the LPARs, provides in vitro and in vivo neuroprotection from agents that affect neurons and brain functions [1,2,3,8,9,24,36]. The neuroprotective molecular mechanism(s) of gintonin are mediated through multiple targets such as anti-oxidative stress and anti-inflammatory effects after LPA1 receptor activations. Pb is one of the most prevalent neurotoxicants and is especially detrimental to the developing mammalian brain by causing motor dysfunctions [26,33]. In the present study, we investigated whether gintonin treatment attenuated Pb-induced histological and functional neurotoxicities in the developing rat cerebellum. We observed that gintonin treatment first reduced blood Pb levels in both dams and offspring. Next, we revealed that gintonin can protect the developing rat cerebellum from Pb-induced cerebellar impairments through various marker proteins, including Purkinje cell integrity, apoptosis-related proteins, GABA synthesis and transport-related proteins, LPA1 and NMDA receptor, oxidative stress-related proteins, and BDNF.

In histological studies, Nissl staining revealed that Pb exposure was detrimental to cerebellar development in the offspring by reducing the number of intact Purkinje cells and increasing the number of degenerating Purkinje cells in the developing cerebellar cortex. Following the co-administration of gintonin with Pb, gintonin protected cerebellar Purkinje cells against Pb-induced cell degeneration; CB immunostaining, a specific marker of Purkinje cells, showed that gintonin treatment alleviated the Pb-induced decrease in CB-immunoreactive Purkinje cells in the cerebellum. Thus, the protective effects of gintonin demonstrated in this study are consistent with the results of previous studies in which ginseng extract is shown to prevent the toxic effects of heavy metals, including Pb, mercury, and cadmium [37,38,39].

In the apoptosis-related evaluation regarding the reduced number of intact Purkinje cells after Pb exposure, we first investigated the expression of Bax, the main regulatory factor in the process of proapoptotic cell death by inducing mitochondrial pore formation, in the developing cerebellum. Pre- and post-natal Pb exposure enhanced the expression of the Bax protein, with Bax-immunoreactive cells mainly observed in the PL of the cerebellum. After gintonin co-administration with Pb, fewer Bax-immunoreactive cells were observed, suggesting that the Pb-mediated Bax protein induction was repressed by gintonin co-treatment. Similarly, in our previous study, we observed that Pb exposure increased the expression of Bax, but ascorbic acid reversed its increase in the developing cerebellum and hippocampus [40,41]. Meanwhile, anti-apoptotic Bcl2 was reduced by Pb, while gintonin treatment increased Bcl2 expression. These results demonstrating the neuroprotective effects of gintonin in the Pb-exposed offspring are also consistent with those of another study evaluating gintonin-mediated anti-apoptosis in a Parkinson’s disease animal model [4].

Next, we investigated the expression of BDNF and Sirt1, important regulators in cerebellar neuron development and survival. In addition, BDNF plays a central neuroprotective role by opposing damage caused by ischemia, traumatic injury, multiple sclerosis, heavy metal intoxication, and aging [18,29,42,43]. Sirt1 is a regulator of the CREB-BDNF signaling, important for neurogenesis and subsequent synapse formation in the developing and adult brain [29,42,44,45,46]. In accordance with a previously reported pattern of change [18,47], BDNF and Sirt1 were reduced following Pb exposure; gintonin ameliorated the Pb-mediated reduction in cerebellar BDNF and Sirt1. Consistent with the result of a previous study [29], gintonin administration reversely increased both BDNF and Sirt1 in the cerebellum. These results indicate that the gintonin-mediated increase in BDNF and Sirt1 could contribute to the attenuation of Bax expression and enhancement of Bcl2 expression and might be further linked to an increase in the survival of cerebellar cells, including Purkinje cells, even after Pb insults.

As shown in Figure 2, Pb exposure not only reduced the number of Purkinje cells but also damaged the complexity of the dendritic branching of Purkinje cells. In addition, Pb exposure impaired axon fibers from Purkinje cells, which are prominent in pup cerebellum [48]. Further, oligodendrocytes play a key role in myelin sheath formation and myelination [49]. Recent studies have shown the harmful effects of Pb including the disruption of oligodendrocyte differentiation and the reduction of oligodendrocytes following Pb exposure [26,45]. Similarly, we observed that long-term Pb exposure reduced MBP, a marker protein of the myelin sheath, and Olig2-immunoreactivity, a marker protein of the oligodendrocyte. Gintonin treatment restored the reduced MBP and Olig2 induced by Pb. Thus, gintonin administration recovered the Pb-induced Purkinje cell body damages, as well as axonal myelin sheath-forming oligodendrocytes and MBP expression.

In behavioral tests, we evaluated the cerebellar function by using the wire mesh ascending test and bar holding test. Importantly, Pb exposure resulted in functional deterioration, with the rats demonstrating a longer latency to reach the top of the ascending wire mesh and shorter time on the bar in the bar holding test. However, gintonin treatment attenuated the Pb-induced functional impairment by improving the time spent in these behavioral tests (Figure 7). Thus, the gintonin-mediated functional amelioration of locomotion after Pb exposure could be attributed to the gintonin-mediated cerebellar cell recovery, along with the increased expression of the anti-apoptotic protein Bcl2, BDNF, and Sirt1, and oligodendrocytes in the developing rat.

In addition to the analyses of cerebellar cell numbers, apoptosis- and survival-related proteins, and axon-related proteins, we evaluated Pb-induced changes in cell membrane proteins such as GAD67, GABAT1, glutamate receptors, and LPA1 receptor in the developing cerebellum, to elucidate the molecular mechanisms underlying the gintonin-mediated cerebellar protection. GAD67 and NMDAR1 were detected in the somata of Purkinje cells and some cells in the ML. Pb exposure significantly reduced the number of GAD67- and NAMDR1-immunoreactive Purkinje cells, while gintonin restored the reduction in the GAD67- and NAMDR1-immunoreactive Purkinje cells. Along with the GABA-synthesizing enzyme, GABAT1-immunoreactive pinceau structures were also affected by the Pb exposure, and gintonin treatment restored the GABAT1-immunoreactive pinceau structures in the same manner as GAD67 in the cerebellum (Figure 5). In the immunoblotting study, the expression levels of synaptophysin, one of the synapse marker proteins, were reduced after Pb exposure; gintonin treatment restored the expression of the synaptophysin protein consistent with the gintonin-mediated synaptic protection shown in the present and previous studies [18,25]. Although further studies are required to comprehensively understand the change in glutamatergic and GABAergic markers, our present results suggest that Pb exposure causes cerebellar maldevelopment by affecting the excitatory glutamatergic and inhibitory GABAergic systems. Furthermore, gintonin administration attenuates Pb exposure-induced insult to synaptic protein integrity.

Further, LPAR1 was mainly detected in the PL and its expression was affected by Pb-induced cerebellar neurotoxicity (Figure 3 and Figure 4). Among the LPAR subtypes, LPA1 might play a key role in brain development including the cerebellum; the LPA–LPAR1 ***axis*** mediates cellular processes, including cell survival, proliferation, differentiation, migration, adhesion, and morphology of the developing brain [50]. In the present study, we observed a decrease in LPAR1-immunoreactive Purkinje cells and protein expression levels in the cerebellum. Notably, co-administration of gintonin with Pb restored LPAR1 immunoreactivity and expression levels, indicating that Pb negatively affects LPAR1 expression during cerebellar development and gintonin reversed these effects. Thus, gintonin-mediated changes in LPAR1 might contribute to the attenuation of Pb-induced neurotoxicity during cerebellar development.

In addition, oxidative stress, one of the mechanisms of the Pb-induced neurotoxicity, is accumulated by the continuously generated reactive oxygen species [51]. In line with the previously reported induction of endogenous antioxidants owing to Pb exposure [25,26], we presently confirmed that cerebellar Mn-SOD expression showed a similar pattern of changes from the Pb-alone and Pb + GT groups. Nrf2 is an important transcription factor regulating the expression of antioxidants [52]. We observed that Pb exposure leads to the upregulation of Nrf2, with gintonin treatment further increasing the level of Nrf2 in the cerebellum. In previous studies with a Parkinson’s disease animal model using MPTP, treatment with MPTP induced degeneration of brain striatum neurons, with a minimal decrease or increase in Nrf2 expression levels; the oral administration of gintonin significantly increased the striatal Nrf2 expression level [4,36]. Thus, the Nrf2 expression level could be differentially regulated by agents that induce oxidative stress. In addition, gintonin could regulate Nef2 expression differentially from Nrf2 expression, which is dependent on the agent and brain regions. Along with oxidative stresses, neuro-inflammation is also an important mechanism of Pb exposure-mediated neurotoxicity [53]. The Pb-alone treatment activated inflammatory pathways and our results reveal that gintonin treatment attenuated Pb-induced upregulation of pro-inflammatory IL-1β and TNFα in the cerebellum.

**Figure 8:**
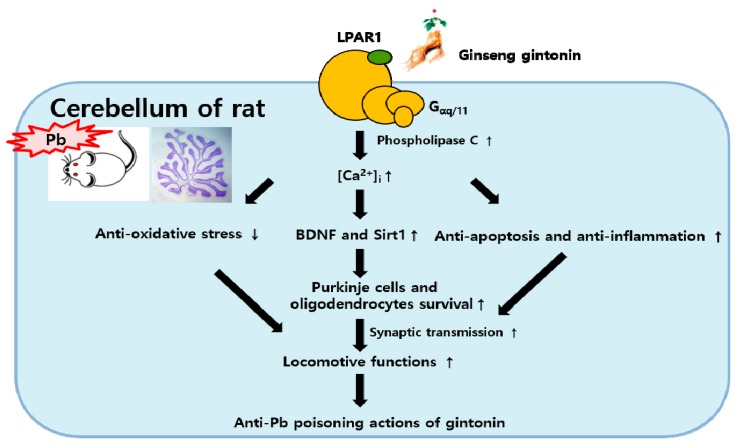
Schematic illustration of gintonin-mediated anti-Pb effects. Cerebellar LPAR1 expression was reduced after Pb exposure, which was reversed by gintonin treatment. Gintonin-mediated anti-Pb effects could be mediated through three mechanisms: antioxidant stress, increased BDNF and Sirt1, and anti-apoptosis/inflammation via activation of LPAR1 signaling pathways. The convergence of these three beneficial mechanisms could increase the survival of cerebellar Purkinje cells and oligodendrocytes, facilitating synaptic transmission. These beneficial effects of gintonin contribute to the locomotive recovery in pups from Pb-poisoned dams.

We demonstrated that long-term Pb administration to dams induced structural and functional cerebellar maldevelopments in the offspring. However, gintonin treatment in dams exposed to Pb increased the survival of cerebellar cells and ameliorated Pb-induced cerebellar malfunctions in the offspring via multiple pathways, including anti-apoptosis, increase in BDNF/Sirt1 expression, and antioxidative stress activity initiated by LPAR1 activation (Figure 8). Thus, we revealed a possibility that gintonin, an active component of ginseng and exogenous LPAR ligand, can be used as a preventive agent against Pb-induced neurotoxicity in the developing brain during pregnancy and lactation.

## 5. Conclusions

The present study demonstrated that Pb administration is toxic to the cerebellum by altering normal development. Pb exposure caused damages by reducing the number of intact Purkinje cells and the expression of synaptophysin, GABA-synthesizing GAD67, GABA transporter 1, BDNF, Sirt1, LPAR1, and MBP, and inducing the expression of glutamatergic NMDAR1, Nrf2, Mn-SOD, IL-1β, and TNFα. Gintonin treatment reduced Pb-induced structural changes. Additionally, gintonin-mediated functional improvements may be owing to structural protections from Pb-induced alterations in cerebellar development. These results highlight the potential use of gintonin as a preventive agent against Pb-induced cerebellar developmental deterioration in pups from mothers at high risk for Pb exposure during pregnancy and lactation.

## References

[1] Kim H.J., Kim D.J., Shin E.J., Lee B.H., Choi S.H., Hwang S.H., Rhim H., Cho I.H., Kim H.C., Nah S.Y. (2016). Effects of gintonin-enriched fraction on hippocampal cell proliferation in wild-type mice and an APPswe/PSEN-1 double Tg mouse model of Alzheimer’s disease. Neurochem. Int..

[2] Cho H.J., Choi S.H., Kim H.J., Lee B.H., Rhim H., Kim H.C., Hwang S.H., Nah S.Y. (2019). Bioactive lipids in gintonin-enriched fraction from ginseng. J. Ginseng Res..

[3] Jang M., Choi J.H., Chang Y., Lee S.J., Nah S.Y., Cho I.H. (2019). Gintonin, a ginseng-derived ingredient, as a novel therapeutic strategy for Huntington’s disease: Activation of the Nrf2 pathway through lysophosphatidic acid receptors. Brain Behav. Immu..

[4] Jo M.G., Ikram M., Jo M.H., Yoo L., Chung K.C., Nah S.Y., Hwang H., Rhim H., Kim M.O. (2019). Gintonin mitigates MPTP-induced loss of nigrostriatal dopaminergic neurons and accumulation of α-synuclein via the Nrf2/HO-1 pathway. Mol. Neurobiol..

[5] Lee M.J., Chang B.J., Oh S., Nah S.Y., Cho I.H. (2018). Korean red ginseng mitigates spinal demyelination in a model of acute multiple sclerosis by downregulating p38 mitogen-activated protein kinase and nuclear factor-κB signaling pathways. J. Ginseng Res..

[6] Leung K.W., Wong A.S.T. (2010). Pharmacology of ginsenosides: a literature review. Chin. Med..

[7] Hwang S.H., Shin T.J., Choi S.H., Cho H.J., Lee B.H., Pyo M.K., Lee J.H., Kang J., Kim H.J., Park C.W. (2012). Gintonin, newly identified compounds from ginseng, is novel lysophosphatidic acids-protein complexes and activates G protein-coupled lysophosphatidic acid receptors with high affinity. Mol. Cells.

[8] Choi S.H., Jung S.W., Lee B.H., Kim H.J., Hwang S.H., Kim H.K., Nah S.Y. (2015). Ginseng pharmacology: a new paradigm based on gintonin-lysophosphatidic acid receptor interactions. Front. Pharmacol..

[9] Nam S.M., Hwang H., Seo M., Chang B.J., Kim H.J., Choi S.H., Rhim H., Kim H.C., Cho I.H., Nah S.Y. (2018). Gintonin attenuates D-galactose-induced hippocampal senescence by improving long-term hippocampal potentiation, neurogenesis, and cognitive functions. Gerontology.

[10] Rice D., Barone S. (2000). Critical periods of vulnerability for the developing nervous system: evidence from humans and animal models. Environ. Health Perspect..

[11] Aghajanian G.K., Bloom F.E. (1967). The formation of synaptic junctions in developing rat brain: a quantitative electron microscopic study. Brain Res..

[12] Ye X., Fukushima N., Kingsbury M.A., Chun J. (2002). Lysophosphatidic acid in neural signaling. Neuroreport.

[13] Castilla-Ortega E., Pedraza C., Chun J., de Fonseca F.R., Estivill-Torrús G., Santín L.J. (2012). Hippocampal c-Fos activation in normal and LPA1-null mice after two object recognition tasks with different memory demands. Behav. Brain Res..

[14] Suckau O., Gross I., Schrötter S., Yang F., Luo J., Wree A., Chun J., Baska D., Baumgart J., Kano K. (2019). LPA1, LPA2, LPA4, and LPA6 receptor expression during mouse brain development. Dev. Dyn..

[15] Parolisi R., Peruffo A., Messina S., Panin M., Montelli S., Giurisato M., Cozzi B., Bonfanti L. (2015). Forebrain neuroanatomy of the neonatal and juvenile dolphin (***T. truncatus*** and ***S. coeruloalba***). Front. Neuroanat..

[16] Kougias D.G., Cortes L.R., Moody L., Rhoads S., Pan Y.X., Juraska J.M. (2018). Effects of perinatal exposure to phthalates and a high-fat diet on maternal behavior and pup development and social play. Endocrinology.

[17] Lanphear B.P. (2015). The impact of toxins on the developing brain. Annu. Rev. Public Health.

[18] Nam S.M., Cho I.S., Seo J.S., Go T.H., Kim J.H., Nahm S.S., Chang B.J., Lee J.H. (2019). Ascorbic acid attenuates lead-induced alterations in the synapses in the developing rat cerebellum. Biol. Trace Elem. Res..

[19] Tanaka T., Abe H., Kimura M., Onda N., Mizukami S., Yoshida T., Shibutani M. (2015). Developmental exposure to T-2 toxin reversibly affects postnatal hippocampal neurogenesis and reduces neural stem cells and progenitor cells in mice. Arch. Toxicol..

[20] World Health Organization (WHO) (2010). Childhood lead poisoning. http://www.who.int/ceh/publications/childhoodpoisoning/en/.

[21] United States Geological Survey (USGS) (2017). Lead. Mineral Commodities Summaries.

[22] Eom S.Y., Lee Y.S., Lee S.G., Seo M.N., Choi B.S., Kim Y.D., Lim J.A., Hwang M.S., Kwon H.J., Kim Y.M. (2018). Lead, mercury, and cadmium exposure in the Korean general population. J. Korean Med. Sci..

[23] Barkur R.R., Bairy L.K. (2016). Histological study on hippocampus, amygdala and cerebellum following low lead exposure during prenatal and postnatal brain development in rats. Toxicol. Ind. Health..

[24] Choi S.H., Jung S.W., Kim H.S., Kim H.J., Lee B.H., Kim J.Y., Kim J.H., Hwang S.H., Rhim H., Kim H.C. (2015). A brief method for preparation of gintonin-enriched fraction from ginseng. J. Ginseng Res..

[25] Nam S.M., Seo J.S., Go T.H., Nahm S.S., Chang B.J. (2019). Ascorbic acid supplementation prevents the detrimental effects of prenatal and postnatal lead exposure on the Purkinje cell and related proteins in the cerebellum of developing rats. Biol. Trace Elem. Res..

[26] Nam S.M., Seo J.S., Nahm S.S., Chang B.J. (2019). Effects of ascorbic acid on osteopontin expression and axonal myelination in the developing cerebellum of lead-exposed rat pups. Int. J. Environ. Res. Public Health.

[27] Takayama C., Inoue Y. (2005). Developmental expression of GABA transporter-1 and 3 during formation of the GABAergic synapses in the mouse cerebellar cortex. Brain Res. Dev. Brain Res..

[28] Nam S.M., Seo J.S., Nahm S.S., Chang B.J. (2019). Effects of ascorbic acid treatment on developmental alterations in calcium-binding proteins and gamma-aminobutyric acid transporter 1 in the cerebellum of lead-exposed rats during pregnancy and lactation. J. Toxicol. Sci..

[29] Nam S.M., Seo M., Seo J.S., Rhim H., Nahm S.S., Cho I.H., Chang B.J., Kim H.J., Choi S.H., Nah S.Y. (2019). Ascorbic acid mitigates D-galactose-induced brain aging by increasing hippocampal neurogenesis and improving memory function. Nutrients.

[30] Whitney E.R., Kemper T.L., Rosene D.L., Bauman M.L., Blatt G.J. (2008). Calbindin-D28k is a more reliable marker of human Purkinje cells than standard Nissl stains: a stereological experiment. J. Neurosci. Methods.

[31] Shen J., Xu L., Qu C., Sun H., Zhang J. (2018). Resveratrol prevents cognitive deficits induced by chronic unpredictable mild stress: Sirt1/miR-134 signalling pathway regulates CREB/BDNF expression in hippocampus in vivo and in vitro. Behav. Brain Res..

[32] Estivill-Torrús G., Llebrez-Zayas P., Matas-Rico E., Santín L., Pedraza C., De Diego I., Del Arco I., Fernández-Llebrez P., Chun J., De Fonseca F.R. (2008). Absence of LPA1 signaling results in defective cortical development. Cereb. Cortex.

[33] Lidsky T.I., Schneider J.S. (2003). Lead neurotoxicity in children: basic mechanisms and clinical correlates. Brain.

[34] Wu C., Sun D. (2015). GABA receptors in brain development, function, and injury. Metab. Brain Dis..

[35] Collin L., Usiello A., Erbs E., Mathis C., Borrelli E. (2004). Motor training compensates for cerebellar dysfunctions caused by oligodendrocyte ablation. Proc. Natl. Acad. Sci. USA.

[36] Choi J.H., Jang M., Oh S., Nah S.Y., Cho I.H. (2018). Multi-target protective effects of gintonin in 1-methyl-4-phenyl-1,2,3,6-tetrahydropyridine-mediated model of Parkinson’s disease via lysophosphatidic acid receptors. Front. Pharmacol..

[37] Mahmoud O.M., Al Badawi M.H., Salem N.A. (2014). Role of Ginseng on mercury chloride-induced testicular lesions in adult albino rat: a histological and immunohistochemical study. EJH.

[38] Shukla R., Kumar M. (2009). Role of Panax ginseng as an antioxidant after cadmium-induced hepatic injuries. Food Chem. Toxicol..

[39] Wang B., Feng G., Tang C., Wang L., Cheng H., Zhang Y., Ma J., Shi M., Zhao G. (2013). Ginsenoside Rd maintains adult neural stem cell proliferation during lead-impaired neurogenesis. Neurol. Sci..

[40] Han J.M., Chang B.J., Li T.Z., Choe N.H., Quan F.S., Jang B.J., Cho I.H., Hong H.N., Lee J.H. (2007). Protective effects of ascorbic acid against lead-induced apoptotic neurodegeneration in the developing rat hippocampus in vivo. Brain Res..

[41] Nam S.M., Chang B.J., Kim J.H., Nahm S.S., Lee J.H. (2018). Ascorbic acid ameliorates lead-induced apoptosis in the cerebellar cortex of developing rats. Brain Res..

[42] Chen F., Zhou C.C., Yang Y., Liu J.W., Yan C.H. (2019). GM1 ameliorates lead-induced cognitive deficits and brain damage through activating the SIRT1/CREB/BDNF pathway in the developing male rat hippocampus. Biol. Trace Elem. Res..

[43] Linker R.A., Lee D.H., Demir S., Wiese S., Kruse N., Siglienti I., Gerhardt E., Neumann H., Sendtner M., Lühder F. (2010). Functional role of brain-derived neurotrophic factor in neuroprotective autoimmunity: therapeutic implications in a model of multiple sclerosis. Brain.

[44] Carter A.R., Chen C., Schwartz P.M., Segal R.A. (2002). Brain-derived neurotrophic factor modulates cerebellar plasticity and synaptic ultrastructure. J. Neurosci..

[45] Ma T., Wu X., Cai Q., Wang Y., Xiao L., Tian Y., Li H. (2015). Lead poisoning disturbs oligodendrocytes differentiation involved in decreased expression of NCX3 inducing intracellular calcium overload. Int. J. Mol. Sci..

[46] Schwartz P.M., Borghesani P.R., Levy R.L., Pomeroy S.L., Segal R.A. (1997). Abnormal cerebellar development and foliation in BDNF-/- mice reveals a role for neurotrophins in CNS patterning. Neuron.

[47] Hossain S., Bhowmick S., Jahan S., Rozario L., Sarkar M., Islam S., Basunia M.A., Rahman A., Choudhury B.K., Shahjalal H. (2016). Maternal lead exposure decreases the levels of brain development and cognition-related proteins with concomitant upsurges of oxidative stress, inflammatory response and apoptosis in the offspring rats. Neurotoxicology.

[48] Toews A.D., Krigman M.R., Thomas D.J., Morell P. (1980). Effect of inorganic lead exposure on myelination in the rat. Neurochem. Res..

[49] Laursen L.S., Chan C.W., Ffrench-Constant C. (2011). Translation of myelin basic protein mRNA in oligodendrocytes is regulated by integrin activation and hnRNP-K. J. Cell Biol..

[50] Sheng X., Yung Y.C., Chen A., Chun J. (2015). Lysophosphatidic acid signalling in development. Development.

[51] Hsu P.C., Liu M.Y., Hsu C.C., Chen L.Y., Guo Y.L. (1997). Lead exposure causes generation of reactive oxygen species and functional impairment in rat sperm. Toxicology.

[52] Kraft A.D., Johnson D.A., Johnson J.A. (2004). Nuclear factor E2-related factor 2-dependent antioxidant response element activation by tert-butylhydroquinone and sulforaphane occurring preferentially in astrocytes conditions neurons against oxidative insult. J. Neurosci..

[53] Metryka E., Chibowska K., Gutowska I., Falkowska A., Kupnicka P., Barczak K., Chlubek D., Baranowska-Bosiacka I. (2018). Lead (Pb) exposure enhances expression of factors associated with inflammation. Int. J. Mol. Sci..

